# Comparison of Medication Prescribing Before and After the COVID-19 Pandemic Among Nursing Home Residents in Ontario, Canada

**DOI:** 10.1001/jamanetworkopen.2021.18441

**Published:** 2021-08-02

**Authors:** Michael A. Campitelli, Susan E. Bronskill, Laura C. Maclagan, Daniel A. Harris, Cecilia A. Cotton, Mina Tadrous, Andrea Gruneir, David B. Hogan, Colleen J. Maxwell

**Affiliations:** 1 ICES (formerly Institute for Clinical Evaluative Sciences), Toronto, Ontario, Canada; 2 Institute of Health Policy, Management and Evaluation, Dalla Lana School of Public Health, University of Toronto, Toronto, Ontario, Canada; 3 Sunnybrook Research Institute, Sunnybrook Health Sciences Centre, Toronto, Ontario, Canada; 4 Women’s College Research Institute, Women’s College Hospital, Toronto, Ontario, Canada; 5 Division of Epidemiology, Dalla Lana School of Public Health, University of Toronto, Toronto, Ontario, Canada; 6 Department of Statistics and Actuarial Science, University of Waterloo, Waterloo, Ontario, Canada; 7 Leslie Dan Faculty of Pharmacy, University of Toronto, Toronto, Ontario, Canada; 8 Department of Family Medicine, University of Alberta, Edmonton, Canada; 9 Division of Geriatric Medicine, Cumming School of Medicine, University of Calgary, Calgary, Alberta, Canada; 10 School of Pharmacy, University of Waterloo, Waterloo, Ontario, Canada; 11 Public Health Sciences, University of Waterloo, Waterloo, Ontario, Canada

## Abstract

**Importance:**

COVID-19 has had devastating effects on the health and well-being of older adult residents and health care professionals in nursing homes. Uncertainty about the associated consequences of these adverse effects on the use of medications common to this care setting remains.

**Objective:**

To examine the association between the COVID-19 pandemic and prescription medication changes among nursing home residents.

**Design, Setting, and Participants:**

This population-based cohort study with an interrupted time-series analysis used linked health administrative data bases for residents of all nursing homes (N = 630) in Ontario, Canada. During the observation period, residents were divided into consecutive weekly cohorts. The first observation week was March 5 to 11, 2017; the last observation week was September 20 to 26, 2020.

**Exposures:**

Onset of the COVID-19 pandemic on March 1, 2020.

**Main Outcomes and Measures:**

Weekly proportion of residents dispensed antipsychotics, benzodiazepines, antidepressants, anticonvulsants, opioids, antibiotics, angiotensin receptor blockers (ARBs), and angiotensin-converting enzyme (ACE) inhibitors. Autoregressive integrated moving average models with step and ramp intervention functions tested for level and slope changes in weekly medication use after the onset of the pandemic and were fit on prepandemic data for projected trends.

**Results:**

Across study years, the annual cohort size ranged from 75 850 to 76 549 residents (mean [SD] age, 83.4 [10.8] years; mean proportion of women, 68.9%). A significant increased slope change in the weekly proportion of residents who were dispensed antipsychotics (parameter estimate [β] = 0.051; standard error [SE] = 0.010; *P* < .001), benzodiazepines (β = 0.026; SE = 0.003; *P* < .001), antidepressants (β = 0.046; SE = 0.013; *P* < .001), trazodone hydrochloride (β = 0.033; SE = 0.010; *P* < .001), anticonvulsants (β = 0.014; SE = 0.006; *P* = .03), and opioids (β = 0.038; SE = 0.007; *P* < .001) was observed. The absolute difference in observed vs estimated use in the last week of the pandemic period ranged from 0.48% (for anticonvulsants) to 1.52% (for antipsychotics). No significant level or slope changes were found for antibiotics, ARBs, or ACE inhibitors.

**Conclusions and Relevance:**

In this population-based cohort study, statistically significant increases in the use of antipsychotics, benzodiazepines, antidepressants, anticonvulsants, and opioids followed the onset of the COVID-19 pandemic, although absolute differences were small. There were no significant changes for antibiotics, ARBs, or ACE inhibitors. Studies are needed to monitor whether changes in pharmacotherapy persist, regress, or accelerate during the course of the pandemic and how these changes affect resident-level outcomes.

## Key Points

**Question:**

How are COVID-19 and related disruptions in care associated with changes in the dispensation of medications commonly used among nursing home residents?

**Findings:**

In this population-based cohort study with an interrupted time-series analysis of all nursing home residents from the 630 facilities in Ontario, Canada, the emergence of the COVID-19 pandemic was associated with significant increases in the use of antipsychotics, benzodiazepines, antidepressants, anticonvulsants, and opioids and no meaningful changes in the use of antibiotics or selected cardiovascular medications.

**Meaning:**

The finding of increased use of medications with the potential for adverse effects among nursing home residents during the initial wave of the pandemic warrants ongoing monitoring for prescribing appropriateness and related resident outcomes.

## Teaser

This population-based cohort study examines the association between the initial wave of the COVID-19 pandemic and changes in medication dispensation patterns among nursing home residents in the province of Ontario, Canada.

## Introduction

The COVID-19 pandemic has had a devastating effect on the quality of care and lives of at-risk older adults.^1,2^ Persons of advanced age with complex chronic conditions, many of whom reside in nursing homes, have high rates of infection^3,4^ and adverse health-related outcomes with COVID-19.^5,6^ In economically advantaged countries, 50% to 80% of COVID-19–related early deaths occurred among older adults in congregate care settings such as nursing homes.^7^ There was widespread transmission of SARS-CoV-2 in these settings, partially owing to structural and operating factors.^8,9,10,11,12^ Resulting workforce shortages and visitor restrictions led to both inadequate staffing^13,14^ and the barring of essential support by family and friends from these homes.^15^ The decline in available clinicians and other health care professionals, along with resident isolation and disruptions in communication, activities, and services during the pandemic, may have resulted in less timely and/or less appropriate clinical care and oversight, including in the management of medications.^16^

A particular concern is that these challenges may have led to a rise in the overuse, misuse, and/or underuse of medications commonly targeted by quality improvement initiatives in the nursing home setting. Although data remain scarce, worries remain about the potential for increased use of psychotropic medications (eg, antipsychotics, benzodiazepines, and antidepressants) among residents during the pandemic, possibly in response to an increase in depression, anxiety, and responsive behaviors coupled with less access to nonpharmacological interventions.^17,18,19^ Using Ontario databases, Stall et al^19^ reported small absolute increases (1.0% to 1.6%) in the mean monthly proportion of nursing home residents dispensed antipsychotics and antidepressants (including trazodone hydrochloride) during the period from March 1 to September 30, 2020, relative to January 1 to February 29, 2020. Beyond psychotropics,^19,20,21,22^ other therapies of interest that have yet to be investigated in this population include opioids, anticonvulsants, and antibiotics.^20,23,24,25,26^ In addition, the initial widespread media reports about the potential risks of angiotensin-converting enzyme (ACE) inhibitors and angiotensin receptor blockers (ARBs) in relation to COVID-19 may have temporarily altered prescribing patterns for these agents among older residents^27,28,29,30^ despite subsequent evidence against these early claims.^31,32,33,34^ A recent US study^35^ reported an initial peak in prescription dispensations from March 15 to 21, 2020, followed by a drop for several medications (including antibiotics), although dispensations declined less and remained more stable for ACE inhibitors and ARBs.

As many jurisdictions look to reform nursing home care after the COVID-19 pandemic, it is important to understand whether and to what extent the pandemic affected medication prescribing for older, frail adults in this setting. Our objective was to comprehensively examine the association between the initial wave of the COVID-19 pandemic and changes in medication dispensation patterns among nursing home residents. We focused on psychotropics, anticonvulsants, opioids, and antibiotics (ie, medication classes potentially subject to inappropriate use in nursing homes) as well as ARBs and ACE inhibitors, the latter in view of possible declines in their use after earlier controversial media reports.

## Methods

### Study Design and Setting

We conducted a population-based cohort study with interrupted time-series analysis of medication use among nursing home residents in Ontario, Canada, from March 5, 2017, to September 26, 2020, using health administrative databases. These databases have been used extensively in previous research^21,22,23,24^ and were linked using unique encoded identifiers and analyzed at ICES (formerly the Institute for Clinical Evaluative Sciences). ICES is an independent, not-for-profit research institute whose legal status under Ontario’s health information privacy law allows it to collect and analyze health care and demographic data, without consent, for health system evaluation and improvement. Our study was authorized under section 45 of Ontario’s Personal Health Information Protection Act,^36^ which does not require review by a research ethics board. Six hundred thirty nursing homes operated in the province during the study period. These facilities provide 24-hour access to nursing and personal care. The Ontario publicly funded provincial health plan covers most nursing home costs and provides residents with universal access to physician services and prescription medications included on the provincial drug program formulary. This study is reported per the Reporting of Studies Conducted Using Observational Routinely Collected Health Data for Pharmacoepidemiology (RECORD-PE) guidelines.^37^

We divided our study period into 186 observation weeks, which started on a Sunday (index date) and ended on a Saturday (first week, March 5-11, 2017; last week, September 20-26, 2020). For each week, we created a cohort of individuals residing in an Ontario nursing home who were alive and eligible to receive medication on the index date. Residence in a nursing home was determined using an algorithm^38^ based on medication claims from the Ontario Drug Benefit database and physician visits from the Ontario Health Insurance Plan database. Specifically, individuals were identified as living in a nursing home on the index date if, in the 90 days before the index date, they had at least 2 Ontario Drug Benefit claims dispensed to a nursing home (separated by ≥7 days) and/or at least 1 physician billing claim to the nursing home setting (eFigure 1 in the Supplement).

On each weekly index date, we excluded residents concurrently admitted to an inpatient facility (0.3%-1.2% of residents across study weeks), without a previous full Resident Assessment Instrument Minimum Data Set, version 2.0, in the Continuing Care Reporting System database (2.7%-3.6% of residents across study weeks), with missing age and/or sex, or 110 years or older (<0.01% of residents across study weeks).

### Data Sources and Measures

Details of the health administrative databases used in this study are provided in eTable 1 in the Supplement. At the index date for each weekly cohort, age, sex, and date of death were obtained from the Ontario Registered Persons database. The Canadian Institute for Health Information (CIHI) Discharge Abstract Database was used to capture concurrent inpatient admissions for residents. In addition, data collected from the most recent full Resident Assessment Instrument Minimum Data Set administration before the index date were used to compute a validated measure of frailty^21,39^ and identify the presence of common comorbidities, including dementia.

We examined various central nervous system medication classes (antipsychotics, benzodiazepines, antidepressants [including trazodone, given its increasing use in nursing homes],^22^ anticonvulsants, and opioids), antibiotics, ARBs, and ACE inhibitors (eTable 2 in the Supplement). For residents in each weekly cohort, we used the Ontario Drug Benefit database to identify those receiving a medication of interest within the observation week based on the dispensation date and days of medication supplied (eFigure 1 in the Supplement).

### Statistical Analysis

Standardized differences were used to compare resident characteristics between the first weekly cohort in March 2020 and each of the first weekly cohorts in March 2017, 2018, and 2019, with a threshold of greater than 10% representing a clinically meaningful difference.^40^ Each medication or class was analyzed separately. We computed the weekly number of individuals dispensed a medication per 100 nursing home residents. Interventional autoregressive integrated moving average (ARIMA) models were used to examine the association of the pandemic with medication use. These models allowed examination of changes in medication prescribing while accounting for serial correlation between consecutive weekly observations (ie, autocorrelation).^41^ Observation weeks from March 1, 2020, and onward were defined as the pandemic period (n = 30); earlier weeks were defined as the prepandemic period (n = 156). We selected March 1, 2020, because COVID-19 cases were evident (or suspected) in Ontario nursing home sites during early March,^42^ and the emergence of these cases would have been expected to gradually limit staffing, family support, and care. To test for both an immediate change in level of prescribing and a slope change in weekly medication use after the onset of the pandemic period, our models included a step intervention function and a ramp intervention function, respectively.^43^ Differencing our weekly data by a lag of 1 week accounted for longitudinal time trends in medication use across the study period and created a stationary series; stationarity was confirmed using augmented Dickey-Fuller tests.^44^ Plots of the autocorrelation function and partial autocorrelation function by weekly lags were used to guide the selection of autoregressive and moving average terms into the ARIMA model; these plots suggested a term was needed to account for an annual trend (52 weeks) when analyzing opioid and antibiotic dispensations (model details are provided in eTable 3 in the Supplement). We examined model fit using autocorrelation function and partial autocorrelation function plots of the model residuals, normality plots of the model residuals, Ljung-Box χ^2^ tests for white noise, and model forecast estimates.

For each medication or class of interest, ARIMA models were also fit to the weeks from the prepandemic period only to forecast the projected weekly proportion of use during observation weeks in the pandemic period. In doing so, we could compare observed weekly medication use during the COVID-19 pandemic with the projected use in the hypothetical absence of a pandemic.

Because psychotropic medications are commonly used and pose specific risks for older residents with dementia,^17,25^ we repeated the ARIMA model analysis among residents with dementia in sensitivity analyses (model details are provided in eTable 4 in the Supplement). Analyses were conducted using SAS, version 9.4 (SAS Institute, Inc). and all statistical tests used 2-sided *P* < .05 as the threshold for significance.

## Results

Across study years, the annual cohort size ranged from 75 850 to 76 549 residents (mean [SD] age, 83.4 [10.8] years; mean proportion of women, 68.9%; mean proportion of men, 31.1%) (Table 1). The distribution of age, sex, frailty, and common comorbidities was similar (standardized differences, <10%) for the first weekly cohort in March 2020 compared with the first weekly cohorts in March 2017, 2018, and 2019. A plot of the weekly number of residents across the study period is presented in eFigure 2 in the Supplement.

**Table 1.** Yearly Baseline Characteristics of Ontario Nursing Home Residents During Study Period^a^

**Characteristic**	**March cohort**				**Standardized difference**^**b**^		
	**2017 cohort (n = 76 275)**^**c**^	**2018 cohort (n = 75 850)**^**d**^	**2019 cohort (n = 76 412)**^**e**^	**2020 cohort (n = 76 549)**^**f**^	**2020 vs 2017**	**2020 vs 2018**	**2020 vs 2019**
Age, mean (SD), y	83.4 (10.8)	83.4 (10.8)	83.4 (10.8)	83.4 (10.8)	0.00	0.00	0.00
Age group, y							
18-64	5131 (6.7)	5044 (6.6)	4973 (6.5)	4960 (6.5)	0.01	0.01	0.00
65-74	8540 (11.2)	8648 (11.4)	9086 (11.9)	9351 (12.2)	0.03	0.03	0.01
75-84	20 441 (26.8)	20 524 (27.1)	20 550 (26.9)	20 650 (27.0)	0.00	0.00	0.00
≥85	42 163 (55.3)	41 634 (54.9)	41 803 (54.7)	41 588 (54.3)	0.02	0.01	0.01
Sex							
Female	52 898 (69.4)	52 462 (69.2)	52 661 (68.9)	52 443 (68.5)	0.02	0.01	0.01
Male	23 377 (30.6)	23 388 (30.8)	23 751 (31.1)	24 106 (31.5)	0.02	0.01	0.01
Frailty							
Robust	10 380 (13.6)	9774 (12.9)	9644 (12.6)	9278 (12.1)	0.04	0.02	0.02
Prefrail	22 327 (29.3)	22 190 (29.3)	22 406 (29.3)	22 924 (29.9)	0.01	0.02	0.01
Frail	43 568 (57.1)	43 886 (57.9)	44 362 (58.1)	44 347 (57.9)	0.02	0.00	0.00
Comorbidities							
Dementia	48 669 (63.8)	48 617 (64.1)	48 649 (63.7)	48 502 (63.4)	0.01	0.02	0.01
Diabetes	20 779 (27.2)	20 703 (27.3)	20 970 (27.4)	21 128 (27.6)	0.01	0.01	0.00
CHF	8493 (11.1)	8463 (11.2)	8514 (11.1)	8558 (11.2)	0.00	0.00	0.00
Hypertension	48 803 (64.0)	48 427 (63.8)	48 811 (63.9)	48 815 (63.8)	0.00	0.00	0.00
Arteriosclerotic heart disease	11 432 (15.0)	11 284 (14.9)	11 363 (14.9)	11 422 (14.9)	0.00	0.00	0.00
Stroke	15 600 (20.5)	15 550 (20.5)	15 375 (20.1)	14 995 (19.6)	0.02	0.02	0.01
Cancer	6326 (8.3)	6331 (8.3)	6612 (8.7)	6789 (8.9)	0.02	0.02	0.01
COPD	11 299 (14.8)	11 111 (14.6)	10 995 (14.4)	10 809 (14.1)	0.02	0.02	0.01
Arthritis	34 184 (44.8)	34 289 (45.2)	33 710 (44.1)	32 987 (43.1)	0.03	0.04	0.02
Depression	25 374 (33.3)	25 081 (33.1)	25 250 (33.0)	25 264 (33.0)	0.01	0.00	0.00
Seizure disorder	4629 (6.1)	4669 (6.2)	4720 (6.2)	4661 (6.1)	0.00	0.00	0.00

Abbreviations: CHF, congestive heart failure; COPD, chronic obstructive pulmonary disease.

^a^ Unless indicated otherwise, data are expressed as No. (%) of patients. Percentages have been rounded and may not total 100.

^b^ A threshold of 0.10 was selected a priori to represent a clinically meaningful difference; no characteristics in any year-to-year comparison reached this threshold.

^c^ Observation week was March 5 to 11, 2017.

^d^ Observation week was March 4 to 10, 2018.

^e^ Observation week was March 3 to 9, 2019.

^f^ Observation week was March 1 to 7, 2020.

**Figure 1.**
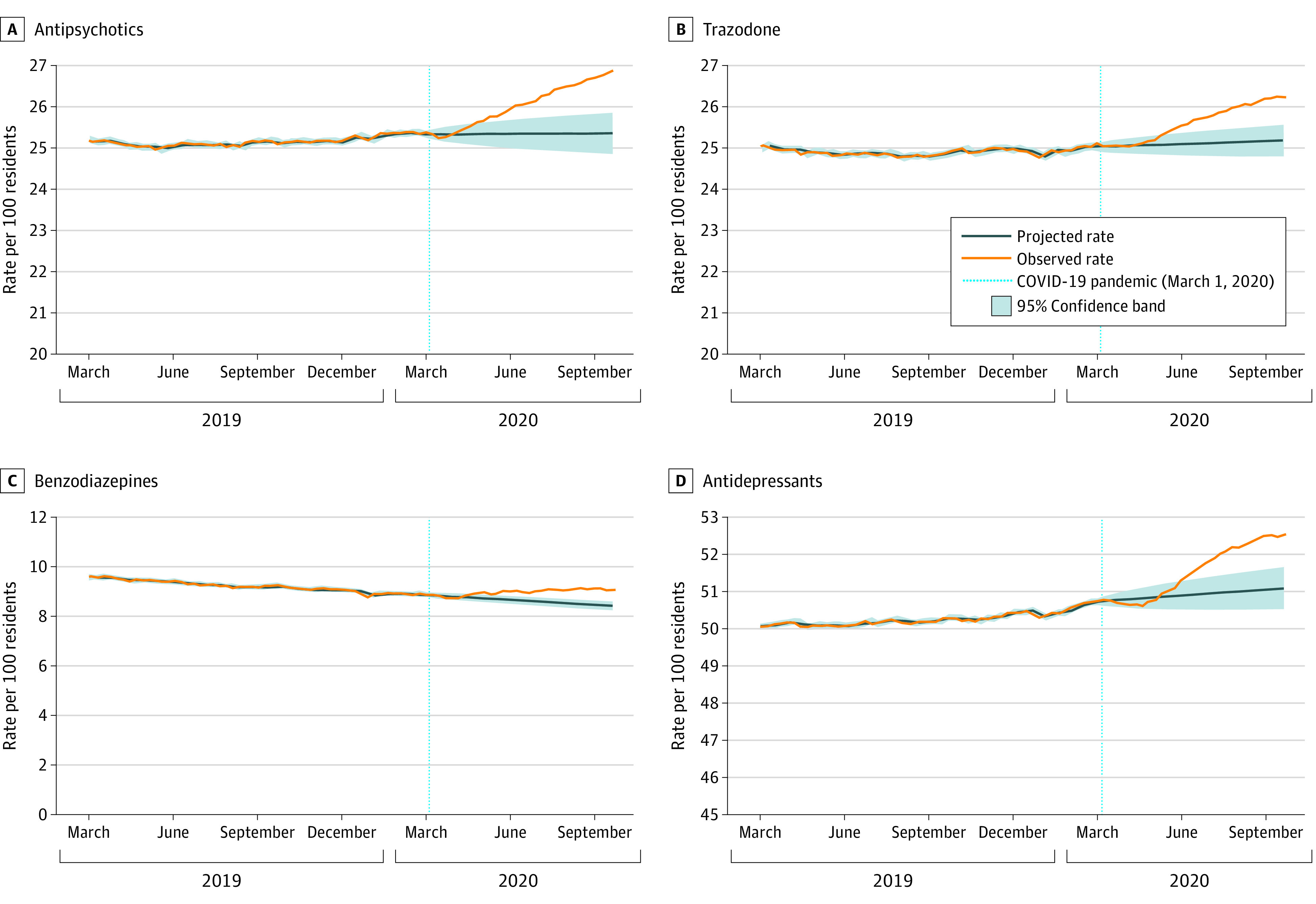
Observed Weekly Proportion of Residents Dispensed Antipsychotics, Trazodone, Benzodiazepines, and Antidepressants Compared With Projected Use Data were plotted from March 2019 to September 2020 to provide greater resolution for trends during the pandemic period.

**Figure 2.**
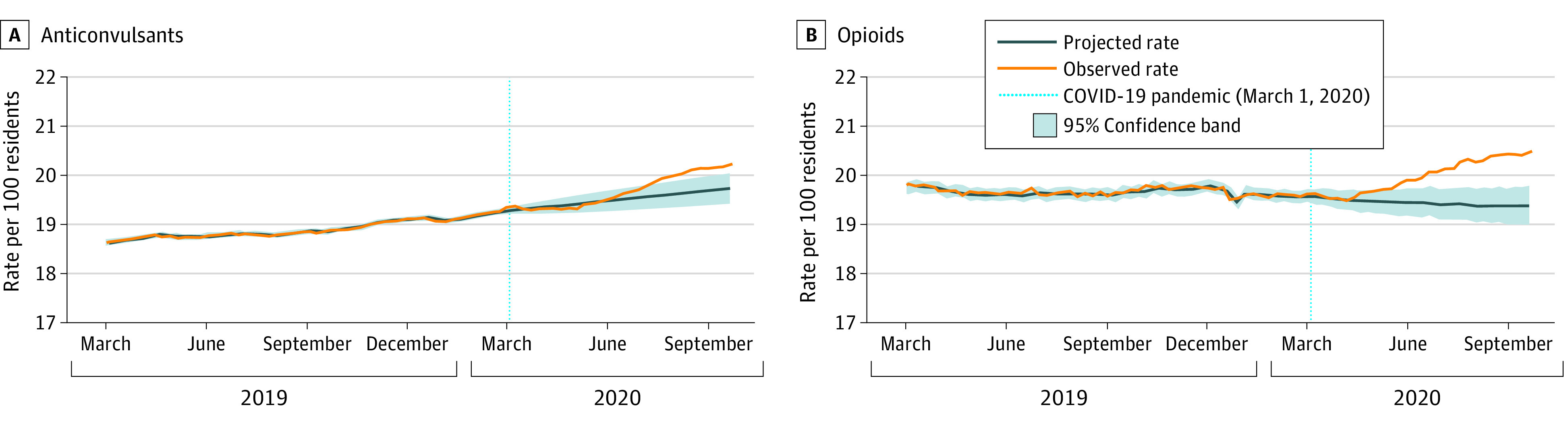
Observed Weekly Proportion of Residents Dispensed Anticonvulsants and Opioids Compared With Projected Use Data were plotted from March 2019 to September 2020 to provide greater resolution for trends during the pandemic period.

**Figure 3.**
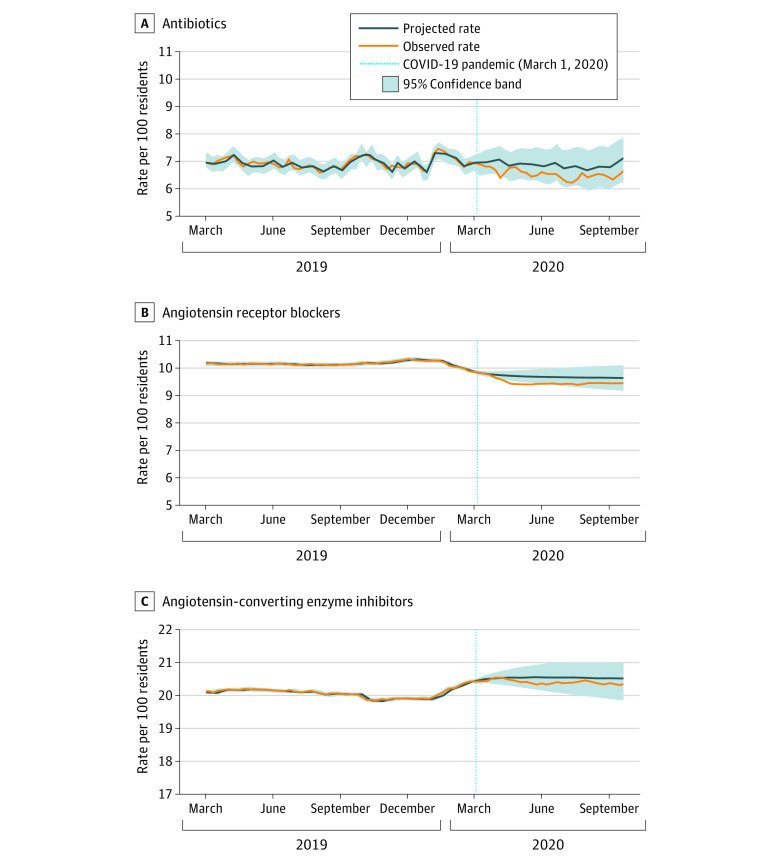
Observed Weekly Proportion of Residents Dispensed Antibiotics, Angiotensin Receptor Blockers (ARBs), and Angiotensin-Converting Enzyme (ACE) Inhibitors Compared With Projected Use Data were plotted from March 2019 to September 2020 to provide greater resolution for trends during the pandemic period.

The overall trends in weekly medication use over time (all classes) are shown in eFigure 3 in the Supplement. Antidepressants (exclusive of trazodone) were the most commonly dispensed medications (52.5% [36 288/69 089 residents in the last study week]), followed by antipsychotics (26.9% [18 563/69 089 residents in the last study week]) and trazodone (26.2% [18 125/69 089 residents in the last study week]). Figure 1, Figure 2, and Figure 3 illustrate trends observed for each medication or class separately (To clearly illustrate observed vs projected estimates after pandemic onset, it was necessary to vary the y-axis). We observed a significant, increased slope change (ramp intervention) among weekly antipsychotic (parameter estimate [β] = 0.051; standard error [SE] = 0.010; *P* < .001), antidepressant (β = 0.046; SE = 0.013; *P* < .001), trazodone (β = 0.033; SE = 0.010; *P* < .001), and anticonvulsant (β = 0.014; SE = 0.006; *P* = .03) use after the onset of the pandemic period (Table 2). There was also a significant, increased slope change among weekly benzodiazepine (β = 0.026; SE = 0.003; *P* < .001) and opioid (β = 0.038; SE = 0.007; *P* < .001) use after the onset of the pandemic, which represented a reversal from the decreasing trends noted for both classes in the prepandemic period. The absolute difference in observed vs projected use in the last week of the pandemic period was highest for antipsychotics (1.52%), followed by antidepressants (1.43%), trazodone (1.06%), opioids (1.06%), benzodiazepines (0.64%), and anticonvulsants (0.48%).

**Table 2.** Autoregressive Integrated Moving Average Model Results Summarizing the Association of the COVID-19 Pandemic With Medication Prescribing in Ontario Nursing Homes

Medication	Prepandemic period^a^		Pandemic period^b^				Step intervention^c^		Ramp intervention^d^	
	Mean (SD) observed weekly proportion of users, %	Observed proportion of users in last week, %^e^	Mean (SD) observed weekly proportion of users, %	Observed proportion of users in last week, %^f^	Estimated proportion of users in last week, %^f^^,^^g^	Absolute difference in observed vs projected users in last week, %^f^^,^^g^	Parameter estimate, β (SE)	*P* value	Parameter estimate, β (SE)	*P* value
Central nervous system										
Antipsychotics	25.20 (0.19)	25.33	26.02 (0.52)	26.87	25.35	1.52	–0.004 (0.045)	.92	0.051 (0.010)	<.001
Benzodiazepines	9.99 (0.70)	8.94	8.99 (0.11)	9.07	8.43	0.64	–0.069 (0.042)	.10	0.026 (0.003)	<.001
Antidepressants	49.59 (0.59)	50.71	51.51 (0.72)	52.52	51.10	1.43	–0.030 (0.051)	.56	0.046 (0.013)	<.001
Trazodone	24.73 (0.23)	25.03	25.60 (0.44)	26.23	25.18	1.06	0.050 (0.049)	.31	0.033 (0.010)	<.001
Anticonvulsants	18.18 (0.67)	19.26	19.67 (0.34)	20.21	19.73	0.48	0.050 (0.030)	.10	0.014 (0.006)	.03
Opioids	20.10 (0.44)	19.56	19.97 (0.34)	20.47	19.40	1.06	–0.050 (0.060)	.40	0.038 (0.007)	<.001
Other										
Antibiotics	7.11 (0.32)	6.96	6.56 (0.18)	6.64	7.07	–0.43	–0.092 (0.127)	.47	–0.011 (0.012)	.38
ARBs	10.08 (0.09)	9.87	9.49 (0.14)	9.44	9.64	–0.20	0.027 (0.025)	.28	–0.005 (0.011)	.63
ACE inhibitors	20.39 (0.36)	20.42	20.41 (0.06)	20.34	20.51	–0.17	–0.007 (0.032)	.83	–0.002 (0.011)	.85

Abbreviations: ACE, angiotensin-converting enzyme; ARBs, angiotensin receptor blockers.

^a^ First observation week of period was March 5 to 11, 2017; last observation week of period, February 23 to 29, 2020.

^b^ First observation week of period was March 1 to 7, 2020; last observation week of period, September 20 to 26, 2020.

^c^ The step intervention tests for an initial immediate level change in weekly medication use after the onset of the pandemic.

^d^ The ramp intervention tests for a change in the slope of weekly medication use in the pandemic period vs the prepandemic period.

^e^ Observation week was February 23 to 29, 2020.

^f^ Observation week was September 20 to 26, 2020.

^g^ The final ARIMA model for each medication class was applied to only observation weeks from the prepandemic period to estimate use in the hypothetical absence of a pandemic.

Both the step and ramp interventions were not statistically significant when analyzing changes in the weekly proportion of antibiotic, ARB, and ACE inhibitor use after the onset of the pandemic. In sensitivity analyses, the magnitude of the slope changes for weekly antipsychotic, antidepressant, trazodone, and benzodiazepine use were reduced but remained statistically significant in our models restricted to residents with dementia (eTable 5 in the Supplement).

## Discussion

We found that the emergence of the COVID-19 pandemic was associated with statistically significant slope increases in the dispensation of antipsychotics, benzodiazepines, antidepressants (including trazodone), anticonvulsants, and opioids among residents of nursing homes in Ontario. In absolute terms, these increases were small compared with projected use (ie, approximately ≤1% for most classes, although slightly higher for antipsychotics and antidepressants). Interestingly, the slope increases and absolute differences in use observed for antipsychotics, antidepressants, and benzodiazepines were less pronounced when the analyses were restricted to residents with dementia. We did not observe any immediate level or slope changes of significance in the use of antibiotics, ARBs, or ACE inhibitors. COVID-19 has had a profound effect on the health of nursing home residents,^7,8,9,10^ staffing, and family caregiver availability.^13,14,15^ The consequences likely included a worsening of social isolation and loneliness^45^ and subsequent increased prevalence of depression and responsive behaviors among residents coupled with fewer available nonpharmacological options for their management. These adverse consequences may have contributed to increased prescribing of select medications among Ontario nursing home residents during the first wave of the pandemic.

The absolute increases we observed for the psychotropic medications after the onset of the pandemic were similar to those reported by Stall et al.^19^ Both studies used the same administrative databases but different methodological approaches (eg, we used ARIMA models to estimate level and slope changes, assessed weekly medication patterns, and incorporated death data in deriving weekly denominators). The consistency of these findings enhances confidence in the nature and magnitude of the medication changes associated with the pandemic. Our sensitivity analyses further illustrate novel findings suggesting that slope increases and absolute differences in the use of psychotropics were smaller after the emergence of the first wave of the pandemic among residents with dementia. A UK study^17^ also showed a relatively lower (0.68%) absolute increase in the proportion of patients with dementia prescribed antipsychotics during the COVID-19 pandemic.

Similar to our findings, claims data for all Ontario residents suggest that the use of antidepressants has increased slightly since the onset of the pandemic.^46^ Although this increased use is likely a marker of greater mental health distress, the use of antidepressants and other psychotropics may pose serious health risks to older nursing home residents (eg, increased confusion, sedation, and falls).^22,47,48,49^ It remains unknown what effect the increase in psychotropic medication use has had on resident-level outcomes. Additional research and continued monitoring are required to fully understand the persistence of these changes and their consequences for residents’ health and well-being.

Data are notably scarce regarding the potential association of COVID-19 with opioid prescribing in nursing homes. In the community, the volume of weekly opioid prescriptions in Ontario decreased after the onset of the pandemic.^46^ In contrast, we observed a significant, albeit small, absolute increase in the proportion of nursing home residents who were dispensed an opioid during the same period. Our findings may reflect an increase in opioid use for end-of life care, pain management (eg, possibly from fall-related injuries because of diminished supervision), and/or the management of responsive behaviors among residents with dementia. The increases in benzodiazepine, opioid, and anticonvulsant use may point to a troubling rise in potentially inappropriate concurrent use of these medications during the early phase of the pandemic.^23^

The COVID-19 pandemic has been identified as a potential threat to antimicrobial stewardship with subsequent risks for antibiotic overuse and greater antimicrobial resistance.^50,51,52^ A review of hospitalized patients with COVID-19 found bacterial coinfection to be relatively infrequent, suggesting most patients do not require antibiotics.^53^ Research indicates that antibiotic prescribing in UK general practices increased by an estimated 7% during the pandemic.^54^ Given the concerns associated with increased antimicrobial resistance among vulnerable older adults and risks for antibiotic-related adverse events among those living in nursing homes with high antibiotic use,^55^ it is reassuring that we found no change in antibiotic dispensations among residents after the onset of the COVID-19 pandemic.

Early in the pandemic, various reports^27,28,29,30^ suggested that ARBs and ACE inhibitors might be associated with increased susceptibility to SARS-CoV-2 infection and worse outcomes for COVID-19 cases. Subsequent research^31,32,33,34^ has demonstrated that these agents do not augment COVID-19–related harms. The finding of no significant change in the dispensation of ARBs and ACE inhibitors after the pandemic among Ontario nursing home residents, despite these initial reports, is important given that the abrupt withdrawal of these medications can be harmful.^29^

### Implications

During the past decade, various targeted strategies have successfully reduced the inappropriate use of antipsychotics, benzodiazepines, and opioids within nursing homes.^20,21,22,23^ Concerns have been raised that the COVID-19 pandemic might potentially reverse these successes because of serious disruptions in staffing, support, and services for residents. Our findings and those of Stall et al^19^ provide preliminary support for these concerns, although the absolute increases in psychotropic and other central nervous system medications during the first wave of the pandemic were small. Although a rise in short-term use of psychotropics may be appropriate because of a greater prevalence of accepted indications and diminished access to nonpharmacological options, studies in other populations^56,57^ show high rates of persistence in the use of these medications after initiation. Periodic medication reviews for residents should target psychotropics prescribed during the pandemic to minimize inappropriate persistence in their use.

### Strengths and Limitations

Key strengths of our study include the use of population-based linked clinical and health administrative data (capturing residents of all Ontario nursing homes), robust analytical methods,^41^ and examination of medications commonly used and/or subject to quality monitoring in the nursing home setting. The major limitation of this work was that we were unable to directly correlate the observed medication changes with COVID-19 infection rates or deaths in these nursing homes. This will be an important area for future investigation. Other limitations include the use of dispensation dates (and days supplied) to identify medication use, which may not reflect whether a medication was taken as directed. Typically, this is not an important limitation, because medication use in Ontario nursing homes is closely supervised by clinicians. However, the effect of the pandemic on staff workload may have resulted in more missed doses for residents, particularly in homes with severe COVID-19 outbreaks. Our data do not allow us to comment on the relevant indications for treatment or its appropriateness at the individual level. Finally, our results provide a system-level overview of pandemic-related changes in medication prescribing across all nursing homes in Ontario and may not correspond to the trends experienced within individual nursing homes.

## Conclusions

In this population-based cohort study of nursing home residents in Ontario, Canada, we observed statistically significant increases in the proportion of residents dispensed psychotropic, anticonvulsant, and opioid medications but no meaningful changes in the dispensation of antibiotics, ARBs, or ACE inhibitors during the first wave of the COVID-19 pandemic. Whether these medication trends continued during the second wave of the pandemic—which has arguably presented even greater challenges for staff, residents, and families—requires further study. The drivers and appropriateness of these medication changes, potential variation in prescribing behaviors across homes and clinical subpopulations, and any consequences for residents’ health and quality of life outcomes are all priority areas for future research. Targeted efforts are required to ensure that the increase in high-risk medications observed after the pandemic does not lead to inappropriate long-term use of these agents among residents.

## Notes

Supplement. **eTable 1.** Description of Ontario Health Administrative Databases **eTable 2.** List of Study Medications **eTable 3.** ARIMA Model Details for Each Medication Analyzed **eTable 4.** ARIMA Model Details for Each Psychotropic Medication Analyzed Among the Subgroup of Residents With Dementia **eTable 5.** ARIMA Model Results Summarizing the Association of the COVID-19 Pandemic With Psychotropic Prescribing Among Residents With Dementia in Ontario Nursing Homes **eFigure 1.** Schematic of the Study Cohort Creation Process **eFigure 2.** Weekly Number of Ontario Nursing Home Residents Across the Study Period **eFigure 3.** Weekly Proportion of Ontario Nursing Home Residents Dispensed Select Medications Before and During the Pandemic, March 2019 to September 2020
