# Supplementary material for: Comparison of Medication Prescribing Before and After the COVID-19 Pandemic Among Nursing Home Residents in Ontario, Canada
Source: JAMA Netw Open. 2021 Aug 2;4(8):e2118441. doi: 10.1001/jamanetworkopen.2021.18441 (PMC8329744; doi:10.1001/jamanetworkopen.2021.18441)
Supplement: Supplement. — eTable 1. Description of Ontario Health Administrative Databases eTable 2. List of Study Medications eTable 3. ARIMA Model Details for Each Medication Analyzed eTable 4. ARIMA Model Details for Each Psychotropic Medication Analyzed Among the Subgroup of Residents With Dementia eTable 5. ARIMA Model Results Summarizing the Association of the COVID-19 Pandemic With Psychotropic Prescribing Among Residents With Dementia in Ontario Nursing Homes eFigure 1. Schematic of the Study Cohort Creation Process eFigure 2. Weekly Number of Ontario Nursing Home Residents Across the Study Period eFigure 3. Weekly Proportion of Ontario Nursing Home Residents Dispensed Select Medications Before and During the Pandemic, March 2019 to September 2020 [file jamanetwopen-e2118441-s001.pdf]

Supplementary Online Content

Campitelli MA, Bronskill SE, Maclagan LC, et al. Comparison of medication prescribing before and after the COVID-19 pandemic among nursing home residents in Ontario, Canada. *JAMA Netw Open*. 2021;4(8):e2118441. doi:10.1001/jamanetworkopen.2021.18441

eTable 1. Description of Ontario Health Administrative Databases

eTable 2. List of Study Medications

eTable 3. ARIMA Model Details for Each Medication Analyzed

eTable 4. ARIMA Model Details for Each Psychotropic Medication Analyzed Among the Subgroup of Residents With Dementia

eTable 5. ARIMA Model Results Summarizing the Association of the COVID-19 Pandemic With Psychotropic Prescribing Among Residents With Dementia in Ontario Nursing Homes

eFigure 1. Schematic of the Study Cohort Creation Process

eFigure 2. Weekly Number of Ontario Nursing Home Residents Across the Study Period

eFigure 3. Weekly Proportion of Ontario Nursing Home Residents Dispensed Select Medications Before and During the Pandemic, March 2019 to September 2020

This supplementary material has been provided by the authors to give readers additional information about their work.

© 2021 Campitelli MA et al. *JAMA Network Open*.

eTable 1. Description of Ontario Health Administrative Databases

DATABASE	DESCRIPTION
Registered Persons Database (RPDB)	Provides basic demographic information (age, sex, area of residence, date of birth, date of death), and eligibility start and end dates, for anyone who has ever received an Ontario health card number (i.e., enrolled in the provincial publicly funded health insurance system). Data are available from April 1, 1991.
Drug Identification Number (DIN)	This file contains a near exhaustive list of Drug Identification Numbers used in Canada from January 1990 forward.
Ontario Drug Benefit (ODB) program	Contains claims for prescription drugs received under the Ontario Drug Benefit program, mainly for those age ≥65 years, nursing home residents and persons receiving social assistance. A special field indicates medications dispensed in the nursing home setting. Data are available from April 1, 1990.
Canadian Institute for Health Information - Discharge Abstract Database (CIHI-DAD)	Contains patient-level data (demographic, diagnoses, procedures) for all admissions to acute care hospitals in Ontario. Data are available from April 1, 1988.
Ontario Health Insurance Plan (OHIP) Claims Database	Contains information on most claims for physician-services (and others) paid for by the Ontario Health Insurance Plan, including for nursing home residents. Includes fee-for-service health care practitioners (primarily physicians) and shadow billings for those paid through non-fee-for-service payment plans. Specific service provision codes identify visits to nursing home residents. Data are available from July 1, 1991.
Canadian Institute for Health Information (CIHI) - Continuing Care Reporting System (CCRS) Database	The CCRS long-term care database contains all mandatory clinical assessments (Resident Assessment Instrument – Minimum Data Set 2.0) performed on nursing home residents in Ontario. Assessments are performed by trained medical personnel and collect information on a wide range of health domains including cognitive patterns, mood and behaviour, psychosocial well-being, performance on activities of daily living and clinical diagnoses. Data are available from April 1, 2009.

© 2021 Campitelli MA et al. *JAMA Network Open*.

eTable 2. List of Study Medications

Medication Class	Medications included
Antipsychotics	amisulpride, aripiprazole, asenapine, chlorpromazine, chlorprothixene, clozapine, flupentixol, fluphenazine, haloperidol, loxapine, lurasidone, mesoridazine, methotrimeprazine, olanzapine, paliperidone, periciazine, perphenazine, pimozide, quetiapine, risperidone, thiopropazate, thioridazine, trifluoperazine, ziprasidone, zuclopenthixol
Benzodiazepines[a]	alprazolam, bromazepam, chlordiazepoxide, clonazepam, clorazepate, diazepam, flurazepam, ketazolam, lorazepam, nitrazepam, oxazepam, temazepam, triazolam
Antidepressants	amitriptyline, amoxapine, bupropion, citalopram, clomipramine, desipramine, doxepin, duloxetine, escitalopram, fluoxetine, fluvoxamine, imipramine, maprotiline, mirtazapine, moclobemide, nefazodone, nortriptyline, paroxetine, phenelzine, protriptyline, sertraline, tranylcypromine, trimipramine, venlafaxine
Other Antidepressants	trazodone
Anticonvulsants	brivaracetam, carbamazepine, clobazam, divalproex, eslicarbazepine, ethosuximide, gabapentin, lacosamide, lamotrigine, levetiracetam, mephenytoin, methsuximide, oxcarbazepine, perampanel, phenobarbital, phensuximide, phenytoin, pregabalin, primidone, rufinamide, stiripentol, topiramate, trientine, valproic acid, vigabatrin, zonisamide
Opioids	codeine, fentanyl, hydromorphone, meperidine, methadone (for pain), morphine, oxycodone
Antibiotics[b]	amikacin, amoxicillin, ampicillin, azithromycin, cefaclor, cefadroxil, cefazolin, cefixime, cefoxitin, cefprozil, ceftazidime, ceftolozane, ceftriaxone, cefuroxime, cephalexin, chloramphenicol, ciprofloxacin, clarithromycin, clindamycin, cloxacillin, daptomycin, doxycycline, ertapenem, erythromycin, fidaxomicin, fosfomycin, gentamicin, levofloxacin, linezolid, metronidazole, minocycline, moxifloxacin, nitrofurantoin, norfloxacin, ofloxacin, penicillin, rifaximin, sulfamethoxazole, tetracycline, tigecycline, tobramycin, trimethoprim, vancomycin
Angiotensin Receptor Blockers[c]	candesartan, eprosartan, irbesartan, losartan, olmesartan, telmisartan, valsartan
ACE inhibitors[c]	benazepril, captopril cilazapril, enalapril, fosinopril, lisinopril, perindopril, quinapril, ramipril, trandolapril

ACE = Angiotensin Converting Enzyme
a - We could not examine non-benzodiazepine receptor agonists (z-drugs) because these are not covered by the Ontario Drug Benefit (ODB) program.
b - Excludes topical antibiotics.
c - Includes medications by themselves or in combination with another medication.

© 2021 Campitelli MA et al. *JAMA Network Open*.

eTable 3. ARIMA Model Details for Each Medication Analyzed

	Model details[a]	
**Medication**	**Autoregressive Lags**	**Moving Average Lags**
Antipsychotics	1	none
Benzodiazepines	None	1, 2, 3
Antidepressants	1, 4	none
Trazodone	1[b]	none
Anticonvulsants	1	none
Opioids	52	1, 2
Antibiotics	52	2, 3
Angiotensin Receptor Blockers	1, 2, 3, 4	none
ACE inhibitors	1, 3, 4	none

ACE = Angiotensin Converting Enzyme

a - All series were differenced by one week (as noted in Methods).

b - For trazodone, the ARIMA model fit to the weeks from the pre-pandemic period only required autoregressive terms at lags 1, 2, and 3.

© 2021 Campitelli MA et al. *JAMA Network Open*.

eTable 4. ARIMA Model Details for Each Psychotropic Medication Analyzed Among the Subgroup of Residents With Dementia

	Model details[a]	
**Medication**	**Autoregressive Lags**	**Moving Average Lags**
Antipsychotics	1	none
Benzodiazepines	1, 2, 3, 4	3
Antidepressants	1, 4	none
Trazodone	1[b]	none

a - All series were differenced by one week (as noted in Methods).

b - For trazodone, the ARIMA model fit to the weeks from the pre-pandemic period only required autoregressive terms at lags 1, 2, and 3.

© 2021 Campitelli MA et al. *JAMA Network Open*.

eTable 5. ARIMA Model Results Summarizing the Association of the COVID-19 Pandemic With Psychotropic Prescribing Among Residents With Dementia in Ontario Nursing Homes

	Pre-pandemic period[a]		Pandemic period[b]				Step intervention[c]		Ramp intervention[d]	
**Medications**	**Average observed weekly proportion of users in period**	**Observed proportion of users in last week of the period[e]**	**Average observed weekly proportion of users in period**	**Observed proportion of users in last week of the period[f]**	**Predicted[g] proportion of users in last week of the period[f]**	**Absolute difference in observed vs. predicted[g] users in last week of the period[f]**	**Parameter estimate (SE)**	**P-value**	**Parameter estimate (SE)**	**P-value**
Psychotropic Agents										
Antipsychotics	28.90	29.22	29.72	30.43	29.26	1.17	-0.030 (0.059)	0.612	0.040 (0.011)	<0.001
Benzodiazepines	7.93	7.18	7.09	7.11	6.79	0.32	-0.111 (0.034)	0.001	0.017 (0.002)	<0.001
Antidepressants	50.92	52.35	52.97	53.75	52.84	0.91	-0.058 (0.054)	0.283	0.030 (0.011)	0.005
Trazodone	28.24	28.82	29.27	29.76	29.05	0.71	0.040 (0.059)	0.497	0.022 (0.011)	0.049

SE = Standard Error

a - First observation week of period: March 5th, 2017 to March 11th, 2017; Last observation week of period: February 23rd to February 29th, 2020

b - First observation week of period: March 1st, 2020 to March 7th 2020; Last observation week of period: September 20th to September 26th, 2020

c – The step intervention tests for an initial immediate level change in weekly medication use following the onset of the pandemic

d – The ramp intervention tests for a change in the slope of weekly medication use in the pandemic period versus the pre-pandemic period

e - Observation week: February 23rd to February 29th, 2020

f - Observation week: September 20th to September 26th, 2020

g - The final ARIMA model for each medication class was applied to only observation weeks from the pre-pandemic period to predict use in the hypothetical absence of a pandemic.

© 2021 Campitelli MA et al. *JAMA Network Open*.

eFigure 1. Schematic of the Study Cohort Creation Process

Uses the first observation week (March 5th, 2017 to March 11th, 2017) as a reference point when showing dates and details of lookbacks for inclusion criteria and medication use (in this example, a resident would be flagged as receiving antidepressants and opioids in this observation week).

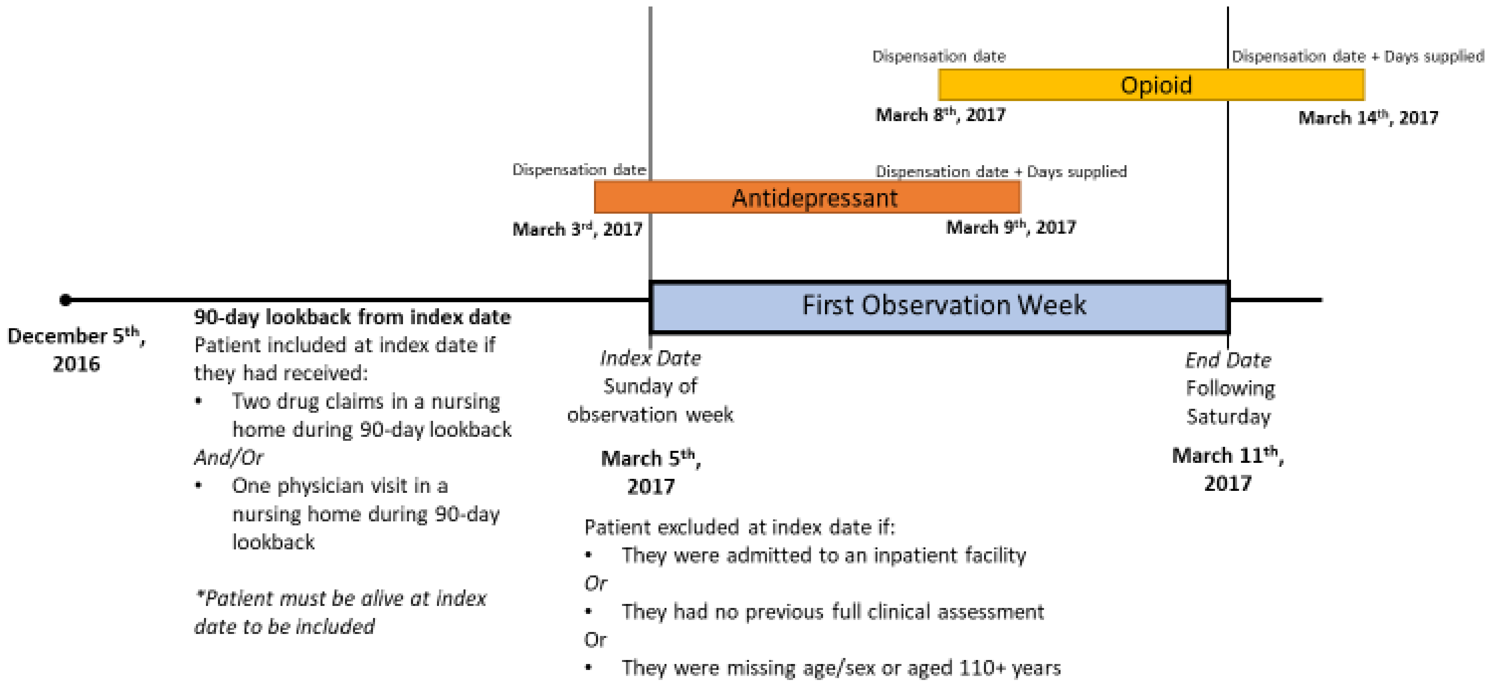

© 2021 Campitelli MA et al. *JAMA Network Open*.

eFigure 2. Weekly Number of Ontario Nursing Home Residents Across the Study Period[a]

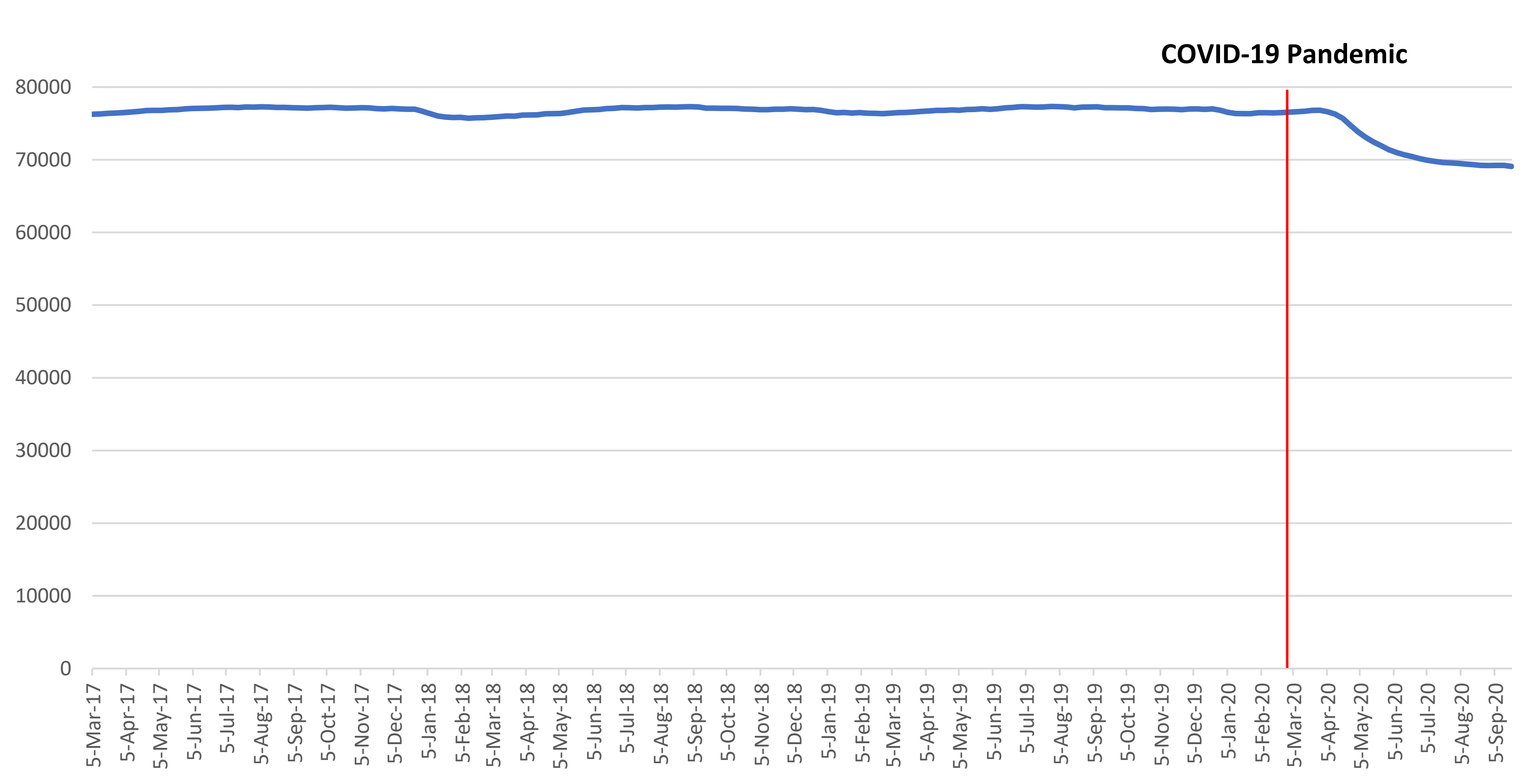

a - The median weekly number of residents during the pre-pandemic and pandemic periods was 76,958 (Interquartile Range [IQR] 76,510-77,151) and 70,856 [IQR 69,506-75,691], respectively.

© 2021 Campitelli MA et al. *JAMA Network Open*.

eFigure 3. Weekly Proportion of Ontario Nursing Home Residents Dispensed Select Medications Before and During the Pandemic, March 2019 to September 2020[a]

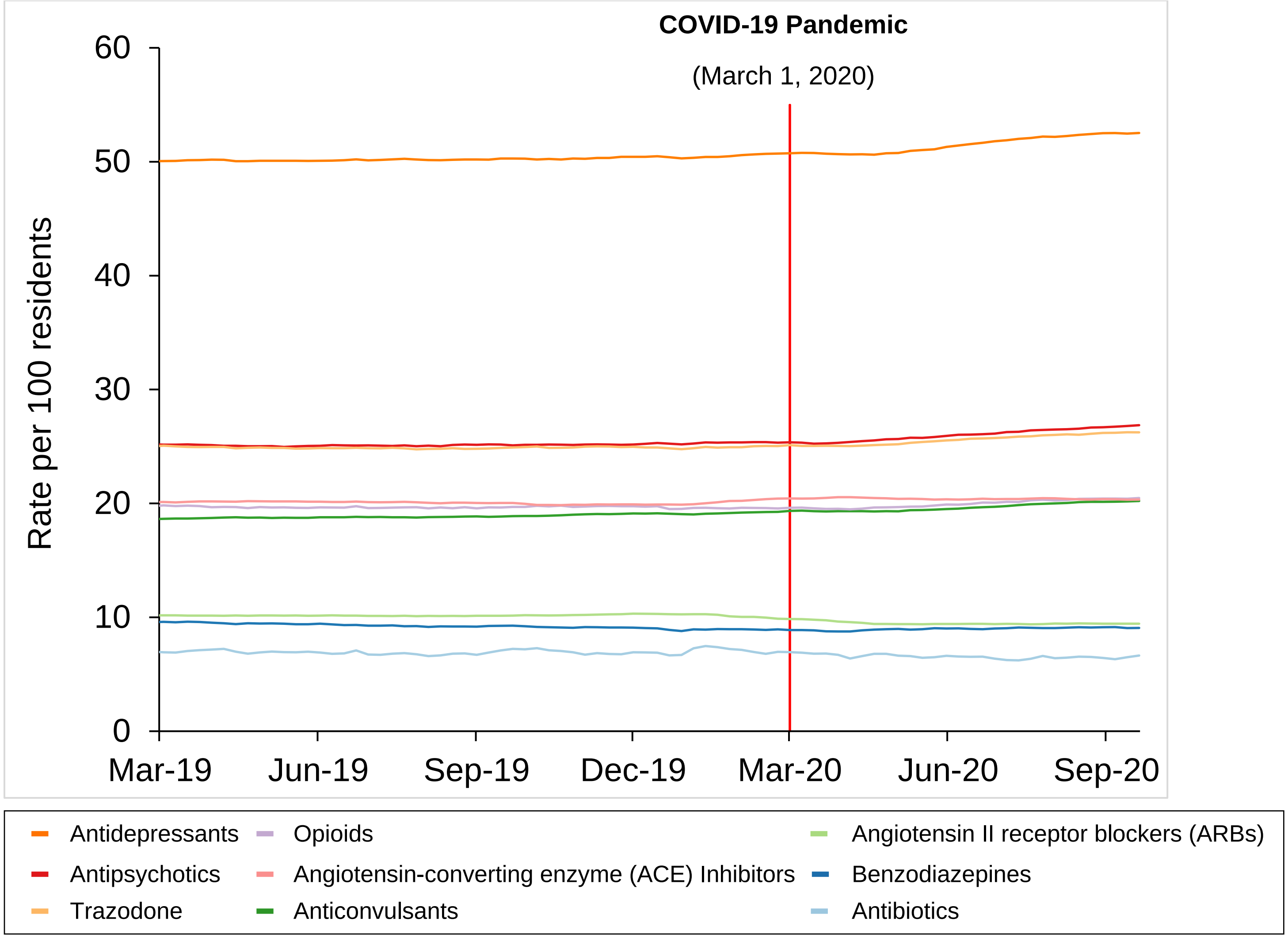

[a]Figure only plotted data from March 2019 onwards to provide greater resolution for trends during the pandemic period.

© 2021 Campitelli MA et al. *JAMA Network Open*.
